# Treatment of a sessile serrated adenoma/polyp deeply invading the appendiceal orifice enabled by combined adaptive traction and underwater endoscopic submucosal dissection

**DOI:** 10.1055/a-2268-5673

**Published:** 2024-03-01

**Authors:** Elena De Cristofaro, Louis-Jean Masgnaux, Alexandru Lupu, Timothée Wallenhorst, Jérémie Jacques, Jérôme Rivory, Mathieu Pioche

**Affiliations:** 1 9318 Gastroenterology Unit, Department of Systems Medicine, University of Rome Tor Vergata, Rome, Italy; 2 Gastroenterology and Endoscopy Unit, Edouard Herriot Hospital, Hospices Civils de Lyon, Lyon, France; 3 Gastroenterology and Endoscopy Unit, Pontchaillou University Hospital, Rennes, France; 4 Gastroenterology and Endoscopy Unit, Dupuytren University Hospital, Limoges, France

Endoscopic submucosal dissection (ESD) of lesions that invade the appendix is technically challenging because of the difficulty of accessing the submucosa. A previous retrospective multicenter study reported a suboptimal R0 resection rate of around 80% 1 . Several tools, such as traction devices, have been developed to facilitate the procedure 2 , including multipolar adaptive traction 3 . We herein report the case of a 73-year-old patient with a large dysplastic sessile serrated adenoma/polyp deeply invading the appendiceal orifice (type 3 of Toyonaga’s classification) 4 , which was successfully resected using an adaptative traction device (A-TRACT 2+2) combined with underwater ESD ( Video 1 ).

Adaptive traction device combined with underwater endoscopic submucosal dissection to treat an appendicular neoplastic lesion.Video 1

After circumferential incision and trimming, the four loops of the A-TRACT 2+2 were fixed by clips to the edges of the lesion, with the appendiceal orifice situated at its center. The rubber band was fixed to the opposite wall. In this way, the dissection was performed with good submucosal exposure circumferentially around the appendiceal area. To remove the entire submucosal attachment of the cecal component, we dissected alternately on the right and on the left. Once the only part remaining was the appendiceal area, the A-TRACT 2+2 was tightened to focus the tension on the appendiceal area enough to bring out the bottom of the appendix, allowing us to cut sideways as deeply as possible ( Fig. 1 ). Underwater ESD was used to optimize the submucosal exposure and go deeper into the very narrow space behind the appendix. With this combined technique, submucosal exposure was good right to the end of the procedure, allowing an R0 resection without adverse events. Histopathological analysis revealed a dysplastic sessile serrated adenoma/polyp with focal intramucosal adenocarcinoma. The defect was closed with endoscopic clips, leaving the appendiceal orifice open in order to avoid appendicitis, as described in strategies for endoscopic full-thickness resection 5 .

**Fig. 1:**
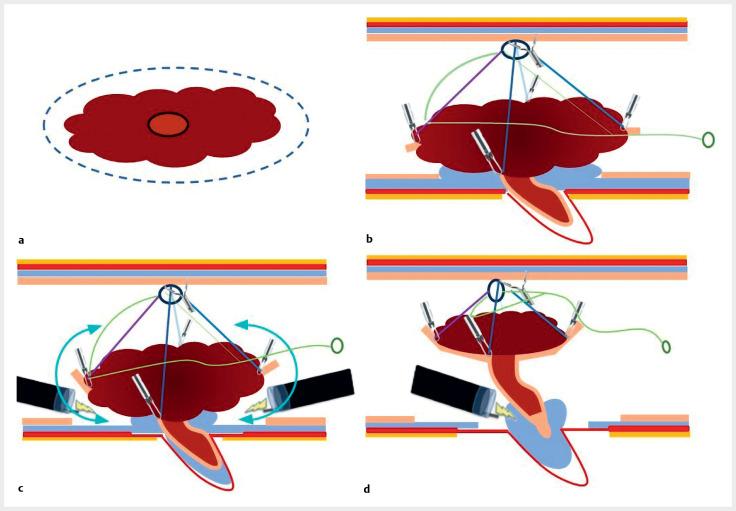
Schematic representation of endoscopic submucosal dissection using the ATRACT 2+2 adaptative traction device. **a** Circumferential incision and trimming; **b** ATRACT 2+2 placement with good submucosal exposure; **c** dissection around the lesion; **d** tightening the ATRACT 2+2 to pull up the bottom of the appendix and cut sideways as deeply as possible.

We hypothesize that a dedicated device of this type combined with underwater ESD could facilitate intervention for appendiceal lesions, especially those deeply invading the appendiceal orifice.

Endoscopy_UCTN_Code_TTT_1AO_2AG

## Notes

Endoscopy E-Videos https://eref.thieme.de/e-videos
*E-Videos* is an open access online section of the journal *Endoscopy* , reporting on interesting cases and new techniques in gastroenterological endoscopy. All papers include a high-quality video and are published with a Creative Commons CC-BY license. Endoscopy E-Videos qualify for HINARI discounts and waivers and eligibility is automatically checked during the submission process. We grant 100% waivers to articles whose corresponding authors are based in Group A countries and 50% waivers to those who are based in Group B countries as classified by Research4Life (see: https://www.research4life.org/access/eligibility/ ). This section has its own submission website at https://mc.manuscriptcentral.com/e-videos .
